# Heterogeneous associations between interleukin-6 receptor variants and phenotypes across ancestries and implications for therapy

**DOI:** 10.1038/s41598-024-54063-3

**Published:** 2024-04-05

**Authors:** Xuan Wang, Molei Liu, Isabelle-Emmanuella Nogues, Tony Chen, Xin Xiong, Clara-Lea Bonzel, Harrison Zhang, Chuan Hong, Yin Xia, Kumar Dahal, Lauren Costa, Jing Cui, VA Million Veteran Program, Sumitra Muralidhar, Jennifer Moser, Jennifer E. Deen, Philip S. Tsao, Sumitra Muralidhar, J. Michael Gaziano, Elizabeth Hauser, Amy Kilbourne, Shiuh-Wen Luoh, Michael Matheny, Dave Oslin, J. Michael Gaziano, Philip S. Tsao, Lori Churby, Stacey B. Whitbourne, Jessica V. Brewer, Shahpoor Shayan, Luis E. Selva, Saiju Pyarajan, Kelly Cho, Scott L. DuVall, Mary T. Brophy, Themistocles L. Assimes, Adriana Hung, Henry Kranzler, Samuel Aguayo, Sunil Ahuja, Kathrina Alexander, Xiao M. Androulakis, Prakash Balasubramanian, Zuhair Ballas, Jean Beckham, Sujata Bhushan, Edward Boyko, David Cohen, Louis Dellitalia, L. Christine Faulk, Joseph Fayad, Daryl Fujii, Saib Gappy, Frank Gesek, Jennifer Greco, Michael Godschalk, Todd W. Gress, Samir Gupta, Salvador Gutierrez, John Harley, Kimberly Hammer, Mark Hamner, Adriana Hung, Robin Hurley, Pran Iruvanti, Frank Jacono, Darshana Jhala, Scott Kinlay, Jon Klein, Michael Landry, Peter Liang, Suthat Liangpunsakul, Jack Lichy, C. Scott Mahan, Ronnie Marrache, Stephen Mastorides, Elisabeth Mates, Kristin Mattocks, Paul Meyer, Jonathan Moorman, Timothy Morgan, Maureen Murdoch, James Norton, Olaoluwa Okusaga, Kris Ann Oursler, Ana Palacio, Samuel Poon, Emily Potter, Michael Rauchman, Richard Servatius, Satish Sharma, River Smith, Peruvemba Sriram, Patrick Strollo, Neeraj Tandon, Philip Tsao, Gerardo Villareal, Agnes Wallbom, Jessica Walsh, John Wells, Jeffrey Whittle, Mary Whooley, Allison E. Williams, Peter Wilson, Junzhe Xu, Shing Shing Yeh, J. Michael Gaziano, Seoyoung C. Kim, Yuk-Lam Ho, Kelly Cho, Tianxi Cai, Katherine P. Liao

**Affiliations:** 1 Department of Population Health Sciences, University of Utah, Salt Lake City, UT USA; 2 Department of Biostatistics, Mailman School of Public Health, Columbia University, New York, NY USA; 3 Department of Biostatistics, Harvard T.H. Chan School of Public Health, Boston, MA 02115 USA; 4 Department of Biomedical Informatics, Harvard Medical School, Boston, MA USA; 5 Division of Rheumatology, Inflammation, and Immunity, Brigham and Women’s Hospital, 60 Fenwood Road, Boston, MA 02115 USA; 6 Department of Biostatistics, Duke University, Durham, NC USA; 7 Department of Statistics and Data Science, Fudan University, Shanghai, China; 8 Massachusetts Veterans Epidemiology Research and Information Center, VA Boston Healthcare System, Boston, MA USA; 9 Division of Aging, Brigham and Women’s Hospital, Boston, MA USA; 10 Division of Pharmacoepidemiology and Pharmacoeconomics, Brigham and Women’s Hospital, Boston, MA USA; 11 Rheumatology Section, VA Boston Healthcare System, Boston, USA; 12 US Department of Veterans Affairs, 810 Vermont Avenue NW, Washington, DC 20420 USA; 13 VA Palo Alto Health Care System, 3801 Miranda Avenue, Palo Alto, CA 94304 USA; 14 VA Boston Healthcare System, 150 S. Huntington Avenue, Boston, MA 02130 USA; 15 Durham VA Medical Center, 508 Fulton Street, Durham, NC 27705 USA; 16 VA HSR&D, 2215 Fuller Road, Ann Arbor, MI 48105 USA; 17 VA Portland Health Care System, 3710 SW US Veterans Hospital Rd, Portland, OR 97239 USA; 18 VA Tennessee Valley Healthcare System, 1310 24th Ave. South, Nashville, TN 37212 USA; 19 Philadelphia VA Medical Center, 3900 Woodland Avenue, Philadelphia, PA 19104 USA; 20 VA Salt Lake City Health Care System, 500 Foothill Drive, Salt Lake City, UT 84148 USA; 21 MVP Coordinating Center, Boston, USA; 22 MVP Coordinating Center, Palo Alto, USA; 23 MVP Information Center, Canandaigua, USA; 24 Canandaigua VA Medical Center, 400 Fort Hill Avenue, Canandaigua, NY 14424 USA; 25 Cooperative Studies Program Clinical Research Pharmacy Coordinating Center, Albuquerque, USA; 26 New Mexico VA Health Care System, 1501 San Pedro Drive SE, Albuquerque, NM 87108 USA; 27 Phoenix VA Health Care System, 650 E. Indian School Road, Phoenix, AZ 85012 USA; 28 South Texas Veterans Health Care System, 7400 Merton Minter Boulevard, San Antonio, TX 78229 USA; 29 Veterans Health Care System of the Ozarks, 1100 North College Avenue, Fayetteville, AR 72703 USA; 30 Columbia VA Health Care System, 6439 Garners Ferry Road, Columbia, SC 29209 USA; 31 William S. Middleton Memorial Veterans Hospital, 2500 Overlook Terrace, Madison, WI 53705 USA; 32 Iowa City VA Health Care System, 601 Highway 6 West, Iowa City, IA 52246-2208 USA; 33 VA North Texas Health Care System, 4500 S. Lancaster Road, Dallas, TX 75216 USA; 34 VA Puget Sound Health Care System, 1660 S. Columbian Way, Seattle, WA 98108-1597 USA; 35 Portland VA Medical Center, 3710 SW U.S. Veterans Hospital Road, Portland, OR 97239 USA; 36 Birmingham VA Medical Center, 700 S. 19th Street, Birmingham, AL 35233 USA; 37 Robert J. Dole VA Medical Center, 5500 East Kellogg Drive, Wichita, KS 67218-1607 USA; 38 VA Southern Nevada Healthcare System, 6900 North Pecos Road, North Las Vegas, NV 89086 USA; 39 VA Pacific Islands Health Care System, 459 Patterson Rd, Honolulu, HI 96819 USA; 40 John D. Dingell VA Medical Center, 4646 John R Street, Detroit, MI 48201 USA; 41 White River Junction VA Medical Center, 163 Veterans Drive, White River Junction, VT 05009 USA; 42 Sioux Falls VA Health Care System, 2501 W 22nd Street, Sioux Falls, SD 57105 USA; 43 Richmond VA Medical Center, 1201 Broad Rock Blvd., Richmond, VA 23249 USA; 44 Hershel “Woody” Williams VA Medical Center, 1540 Spring Valley Drive, Huntington, WV 25704 USA; 45 VA San Diego Healthcare System, 3350 La Jolla Village Drive, San Diego, CA 92161 USA; 46 Edward Hines, Jr. VA Medical Center, 5000 South 5th Avenue, Hines, IL 60141 USA; 47 Cincinnati VA Medical Center, 3200 Vine Street, Cincinnati, OH 45220 USA; 48 Fargo VA Health Care System, 2101 N. Elm, Fargo, ND 58102 USA; 49 Ralph H. Johnson VA Medical Center, Mental Health Research, 109 Bee Street, Charleston, SC 29401 USA; 50 VA Tennessee Valley Healthcare System, 1310 24th Avenue, South Nashville, TN 37212 USA; 51 W.G. (Bill) Hefner VA Medical Center, 1601 Brenner Ave, Salisbury, NC 28144 USA; 52 Hampton VA Medical Center, 100 Emancipation Drive, Hampton, VA 23667 USA; 53 VA Northeast Ohio Healthcare System, 10701 East Boulevard, Cleveland, OH 44106 USA; 54 Louisville VA Medical Center, 800 Zorn Avenue, Louisville, KY 40206 USA; 55 Southeast Louisiana Veterans Health Care System, 2400 Canal Street, New Orleans, LA 70119 USA; 56 VA New York Harbor Healthcare System, 423 East 23rd Street, New York, NY 10010 USA; 57 Richard Roudebush VA Medical Center, 1481 West 10th Street, Indianapolis, IN 46202 USA; 58 Washington DC VA Medical Center, 50 Irving St, Washington, DC 20422 USA; 59 Charles George VA Medical Center, 1100 Tunnel Road, Asheville, NC 28805 USA; 60 VA Maine Healthcare System, 1 VA Center, Augusta, ME 04330 USA; 61 James A. Haley Veterans’ Hospital, 13000 Bruce B. Downs Blvd, Tampa, FL 33612 USA; 62 VA Sierra Nevada Health Care System, 975 Kirman Avenue, Reno, NV 89502 USA; 63 Central Western Massachusetts Healthcare System, 421 North Main Street, Leeds, MA 01053 USA; 64 Southern Arizona VA Health Care System, 3601 S 6th Avenue, Tucson, AZ 85723 USA; 65 James H. Quillen VA Medical Center, Corner of Lamont & Veterans Way, Mountain Home, TN 37684 USA; 66 VA Long Beach Healthcare System, 5901 East 7th Street, Long Beach, CA 90822 USA; 67 Minneapolis VA Health Care System, One Veterans Drive, Minneapolis, MN 55417 USA; 68 VA Health Care Upstate New York, 113 Holland Avenue, Albany, NY 12208 USA; 69 Michael E. DeBakey VA Medical Center, 2002 Holcombe Blvd, Houston, TX 77030 USA; 70 Salem VA Medical Center, 1970 Roanoke Blvd, Salem, VA 24153 USA; 71 Miami VA Health Care System, 1201 NW 16th Street, 11 GRC, Miami, FL 33125 USA; 72 Manchester VA Medical Center, 718 Smyth Road, Manchester, NH 03104 USA; 73 VA Eastern Kansas Health Care System, 4101 S 4th Street Trafficway, Leavenworth, KS 66048 USA; 74 St. Louis VA Health Care System, 915 North Grand Blvd, St. Louis, MO 63106 USA; 75 Syracuse VA Medical Center, 800 Irving Avenue, Syracuse, NY 13210 USA; 76 Providence VA Medical Center, 830 Chalkstone Avenue, Providence, RI 02908 USA; 77 Eastern Oklahoma VA Health Care System, 1011 Honor Heights Drive, Muskogee, OK 74401 USA; 78 N. FL/S. GA Veterans Health System, 1601 SW Archer Road, Gainesville, FL 32608 USA; 79 VA Pittsburgh Health Care System, University Drive, Pittsburgh, PA 15240 USA; 80 Overton Brooks VA Medical Center, 510 East Stoner Ave, Shreveport, LA 71101 USA; 81 VA Palo Alto Health Care System, 3801 Miranda Avenue, Palo Alto, CA 94304-1290 USA; 82 New Mexico VA Health Care System, 1501 San Pedro Drive, S.E. Albuquerque, NM 87108 USA; 83 VA Greater Los Angeles Health Care System, 11301 Wilshire Blvd, Los Angeles, CA 90073 USA; 84 Edith Nourse Rogers Memorial Veterans Hospital, 200 Springs Road, Bedford, MA 01730 USA; 85 Clement J. Zablocki VA Medical Center, 5000 West National Avenue, Milwaukee, WI 53295 USA; 86 San Francisco VA Health Care System, 4150 Clement Street, San Francisco, CA 94121 USA; 87 Bay Pines VA Healthcare System, 10,000 Bay Pines Blvd, Bay Pines, FL 33744 USA; 88 Atlanta VA Medical Center, 1670 Clairmont Road, Decatur, GA 30033 USA; 89 VA Western New York Healthcare System, 3495 Bailey Avenue, Buffalo, NY 14215-1199 USA; 90 Northport VA Medical Center, 79 Middleville Road, Northport, NY 11768 USA

**Keywords:** Genetics, Genetic association study

## Abstract

The Phenome-Wide Association Study (PheWAS) is increasingly used to broadly screen for potential treatment effects, e.g., *IL6R* variant as a proxy for IL6R antagonists. This approach offers an opportunity to address the limited power in clinical trials to study differential treatment effects across patient subgroups. However, limited methods exist to efficiently test for differences across subgroups in the thousands of multiple comparisons generated as part of a PheWAS. In this study, we developed an approach that maximizes the power to test for heterogeneous genotype–phenotype associations and applied this approach to an *IL6R* PheWAS among individuals of African (AFR) and European (EUR) ancestries. We identified 29 traits with differences in *IL6R* variant-phenotype associations, including a lower risk of type 2 diabetes in AFR (OR 0.96) vs EUR (OR 1.0, p-value for heterogeneity = 8.5 × 10^–3^), and higher white blood cell count (p-value for heterogeneity = 8.5 × 10^–131^). These data suggest a more salutary effect of IL6R blockade for T2D among individuals of AFR vs EUR ancestry and provide data to inform ongoing clinical trials targeting IL6 for an expanding number of conditions. Moreover, the method to test for heterogeneity of associations can be applied broadly to other large-scale genotype–phenotype screens in diverse populations.

## Introduction

Large-scale biobanks linked to electronic health records (EHR) offer a promising approach to screen for potential treatment effects^1,2^. In some cases, genetic variants are linked with altered protein expression resulting in an effect similar to a treatment^3^. One example is a missense variant in IL6R Asp(258)Ala, known to reduce membrane-bound IL6R expression and thus dampen IL-6 signaling^4^. The targeted therapies, tocilizumab and sarilumab, block the IL6R pathway. In a phenotypic screen performed in a Phenome-Wide Association Study (PheWAS), subjects carrying the Asp(258)Ala variant were found to have a phenotypic profile similar to those on drugs that block IL-6R; subjects with the IL6R variant have higher hemoglobin and lower high sensitivity C-reactive protein (CRP) compared to those without the variant^5,6^. The PheWAS is a study design in which the association between single-nucleotide polymorphisms or other types of genomic variants are tested for association across a broad range of phenotypes^7^. Thus, population-based biobanks may also provide an opportunity to query potential effects of treatments using genetic variants across a more diverse population than clinical trials. While large amounts of data are now available, limited methods exist to efficiently test for potential heterogeneity across subpopulations in large scale screens such as the PheWAS.

To expand upon the use of biobanks in generating evidence on treatment effects, the PheWAS is a promising approach that can systematically test for associations between a functional genetic variant which mimics the effect of a pharmaceutical agent with a wide spectrum of phenotypes^5,8,9^. Analyses may be stratified by genetic population strata to study heterogenous genotype–phenotype associations which may inform differential treatment effects across populations. Since a large number of hypotheses are being tested at the same time, correction for multiple testing must be undertaken. Traditional methods of multiple testing require large sample sizes, especially when detecting heterogeneity, or group effects from relatively weak signals such as genetic associations. Prior studies have deployed a modified Benjamini–Hochberg procedure (BHq)^10–13^ on the high dimensional heterogeneity or group effect test statistics for selective inference with false discovery rate (FDR) control. In the presence of imbalanced sample sizes across the subgroups, the power of this strategy could be largely impacted by the small sample sizes of the minority groups.

Recent work in adaptive multiple testing enables researchers to construct auxiliary statistics^14^ to increase the power. In one approach, multiple testing of two-sample mean differences with a high dimensional sparse structure used the overall mean statistics as auxiliary information to boost the power^15,16^. In this study, we build upon a new method for false discovery rate (FDR) controlled heterogeneity testing (hetFDR) under a more complicated PheWAS setup with two imbalanced subgroups.

To demonstrate the utility of our proposed hetFDR approach in discovering heterogeneous signals that correspond to potential differential treatment effects, we performed hetFDR on results from a PheWAS with the interleukin-6 receptor (*IL6R*) single nucleotide polymorphism (SNP) (rs2228145, Asp(358)Ala). This variant was selected for several reasons. First, it has been previously studied as a model for IL6R blockade^17,18^. Second, the functional impact of this variant, reduced IL6R expression has also been described^4,19^ where subjects with this variant have lower CRP, higher hemoglobin, and higher levels of soluble IL6R, changes also observed in subjects who receive IL6R blockade^17,20,21^. As well, known therapies exist for control of inflammatory conditions such as rheumatoid arthritis and giant cell vasculitis. More recently, IL-6 blockade has been used for the treatment of hospitalized COVID-19 and with ongoing studies blocking the IL-6 pathway to reduce cardiovascular disease in the general population. Clinical trials remain the gold standard for studying treatment effects but have known limitations in generalizing results to a more diverse population.

The objective was to develop and apply an approach to systematically identify potential heterogeneous genotype–phenotype associations in African (AFR) compared to European (EUR) populations, the two largest ancestries in a diverse mega-biobank cohort, as part of an *IL6R* PheWAS. We hypothesize that this large-scale screen will identify differential effects of the *IL6R* variant across phenotypes with implications for current and future trials targeting the IL6 pathway. Findings were validated in two independent biobank cohorts.

## Materials and methods

### Study design

We performed an *IL6R* PheWAS in the Veterans Affairs Million Veteran Program (MVP) cohort with data up to 09/30/2020. The VA MVP is a longitudinal, multi-institutional cohort study that collects clinical electronic health record (EHR) data, namely inpatient and outpatient data combined with genomic data from participants in approximately 50 Veterans Affairs facilities across the United States. Subjects were included in the MVP if they were 18 years of age or older; had a valid mailing address (to ensure the possibility of follow-up); were able to provide informed consent at the time of recruitment. All participants were required to provide written informed consent upon recruitment. They were asked to (1) complete baseline and lifestyle questionnaires, providing information such as self-reported race/ethnicity, dietary habits, and smoking/drinking status, as well as (2) provide blood samples for genotyping and biomarker studies.

### Statistical analysis

#### PheWAS analysis for each ancestry

The PheWAS analysis was performed using a standardized published approach^22^. Briefly, we fitted a logistic regression for PheWAS analysis to test for association with phenotypes as defined by PheCodes and linear regression for the laboratory analysis. Since many of the laboratory measurements were highly skewed, we tested for association of the *IL6R* variant with log-transformed laboratory values. All models were adjusted for patient age, sex, length of EHR follow up, and health care utilization as measured by the log-total number of PheCodes.

Genetic ancestry was ascertained using previously published methods. Briefly, we trained a logistic regression classification algorithm using self-reported race as silver standard labels and 127 ancestry informative SNPs^23^. The cut-off of predicted probabilities for classification is chosen to guarantee sensitivity is above 0.975. We excluded related MVP participants (halfway between second-degree and third-degree relatives or closer) as measured by the Kinship-Based Inference for GWAS software (https://www.kingrelatedness.com/)^24^. We stratified all association analyses of the *IL6R* variant, rs2228145 (minor allele C; Asp358Ala), with disease phenotypes and laboratory test results by the predicted ancestry group. We focused the analyses on the two largest ancestry groups in MVP, African (AFR) and European (EUR) ancestry.

Within each ancestry group, we performed PheWAS analyses including 1875 phenotypes as defined by PheCodes^25^ and 69 routine laboratory measurements curated in prior studies at the VA, which includes complete blood count and lipid profiles. For each phenotype, a participant was defined as having the condition if they had at least 2 PheCodes, which is often recommended to attain a higher positive predictive value^26^. We excluded PheCodes with a prevalence of 0.5% or less from the analysis and excluded integer level (parent) PheCodes for which corresponding descendant PheCodes already existed, leaving a total of 660 remaining phenotypes. For example, we excluded the integer PheCode 250 (Diabetes mellitus) but included the descendant PheCodes such as 250.1 (Type 1 Diabetes) and 250.2 (Type 2 Diabetes). The screen was also performed on 69 adjudicated laboratory measurements available at VA. Values were defined by the median of all available measurements for each patient. A detailed list of the laboratory tests is in the Supplementary Materials Table S6. We first compared associations between *IL6R* and PheCodes and separately for the curated laboratory values in AFR vs EUR. Significant PheCodes/labs within each ancestry were determined with a false discovery rate (FDR) < 0.1 using the Benjamini–Hochberg procedure (BHq)^13^.

#### Heterogeneity testing with FDR control

Heterogeneity testing was conducted to identify phenotypes and laboratory values to detect a differential association between *IL6R* and phenotype among AFR vs EUR ancestries. To adjust for multiple testing, we developed a novel false discovery rate (FDR) controlled heterogeneity testing (hetFDR) procedure which leverages information from both the mean effect and the magnitude of heterogeneity under a prior assumption that heterogeneous effects are more likely to be present for phenotypes with non-zero mean effects across a large number of candidate phenotypes. The hetFDR procedure is a three-step procedure. In Step (I), for each phenotype, we construct (i) an overall mean effect test statistic as an inverse-variance weighted average effect estimate combining the regression coefficients (against the genetic variant of interest) from the two ancestry groups along with its associated p value; as well as (ii) a chi-square test statistic ascertaining the heterogeneity between the effects as observed from the regression coefficients of the two groups. The mean effect statistic and the heterogeneity statistic are designed to be asymptotically independent so that the validity of tests is ensured when incorporating the mean effect statistics to assist the heterogeneity testing. In Step (II), we use the mean effect statistics to weight the heterogeneity p values, assigning higher prior probabilities of null hypothesis rejection to those phenotypes with more significant mean effects, which corresponds to our prior assumption that phenotypes with non-zero mean effects are more likely to show heterogeneity across the considered ancestry groups. The weighting function is decided adaptively from the data through a regression-based approach. In the final Step (III), we adopt the multiple testing procedure of^27^ on the weighted heterogeneity p-values for detection with FDR control.

Simulation results were conducted, and showed that under different settings of the sample sizes, the heterogeneous effect magnitude, and the number of heterogeneous effects, our proposed hetFDR method controls FDR below 0.1 and shows substantial and consistent higher average power than the existing BHq^13^ and Storey’s procedures^28^. For example, when the sample size of the minority group is 25% of the majority group and the number of phenotypes with heterogeneous effects is 10 out of the totally 50 active ones, our method attains 0.4 higher power than the BHq and Storey’s procedures. Such power gain is also achieved in other settings of different numbers or magnitudes of heterogeneous signals. This is because our method leverages the mean effect statistics as additional information and assigns a higher chance of rejection to the phenotypes with a non-zero mean effect. In addition, the power improvement of our method is more significant in the setting with imbalanced sample sizes between the two ancestry groups, compared to one with equal sample sizes. This is a consequence of having more informative mean effect statistics when one group is larger than the other. A detailed description for the statistical method of heterogeneity multiple testing and the simulation studies are provided in the Supplementary Materials: Statistical Methodology.

### Replication of laboratory results using UK Biobank and MGB Biobank Data

Findings were replicated in UK Biobank (UKB) and the Mass General Brigham (MGB) Biobank^2,29,30^. The UKB is a longitudinal cohort study that prospectively recruits patients to determine the effects of lifestyle, environmental, and genomic factors on disease outcomes over time. The study population includes approximately 500,000 volunteers recruited from the United Kingdom’s general population from 2006 to 2010. Measurements of 61 laboratory biomarkers and blood cell counts were ascertained for all UKB participants as part of a standardized baseline assessment. The MGB Biobank contains linked EHR, and genetic data anchored by two large tertiary care hospitals: Brigham and Women’s Hospital and Massachusetts General Hospital in Boston. The MGB Biobank data consist of 59,052 participants with both EHR data and genomic data available. Laboratory test results were extracted for these patients.

To validate heterogeneous *IL6R*-phenotype associations in AFR vs EUR observed in MVP, we performed analyses in UKB and MGB Biobank data. Due to the relatively smaller size of AFR in these cohorts, the analyses focused on traits with continuous values, i.e., laboratory results.

This study obtained institutional review board approval through the Veterans Affairs MVP under Central IRB #16-06 with title: Cardiovascular Disease Risk Factors, Prevalent Cardiovascular Disease, and Genetics in the Million Veteran Program, and the Mass General Brigham Institutional Review Board. All experiments were performed in accordance with relevant guidelines and regulations. All analyses were performed using R software. The code for analyzing the data is available on GitHub, https://github.com/wx202/HeterTestIL6R.git.

## Results

In the MVP cohort, a total of 545,147 Veterans were included in the analysis, of which 91.3% were male, with a mean (SD) age of 62.1 (13.9) years and a mean (SD) follow-up time of 12.5 (5.7) years. Among these participants, 105,838 were classified as AFR and 439,309 were classified as EUR. In this study, we controlled for an FDR of 10%, which ensures that among the associations considered significant, at most 10% of the associations were false positives^13^. The frequency of the rs2228145 allele in MVP was 14% in AFR and 40% in EUR, in the UKB cohort was 16% in AFR and 41% in EUR, and in the MGB cohort was 17% in AFR and 40% in EUR.

**Figure 1:**
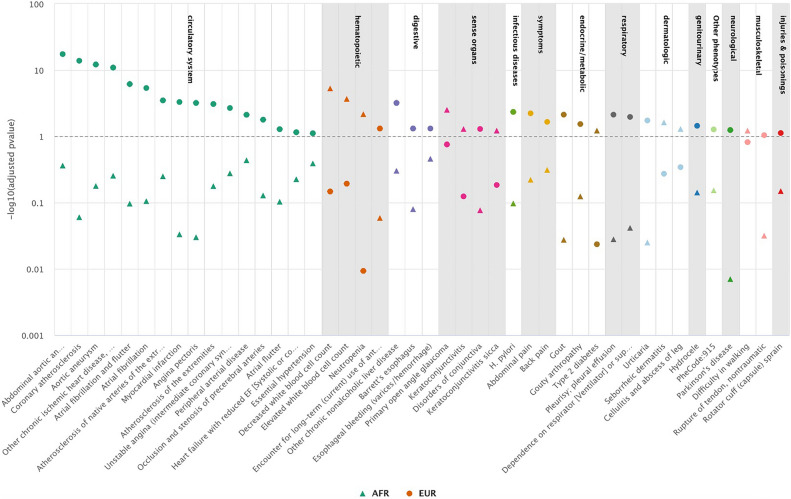
Phenotypes (phecodes) significantly associated with the *IL6R* variant in AFR or EUR (BH adjusted p value ≤ 0.1).

**Figure 2:**
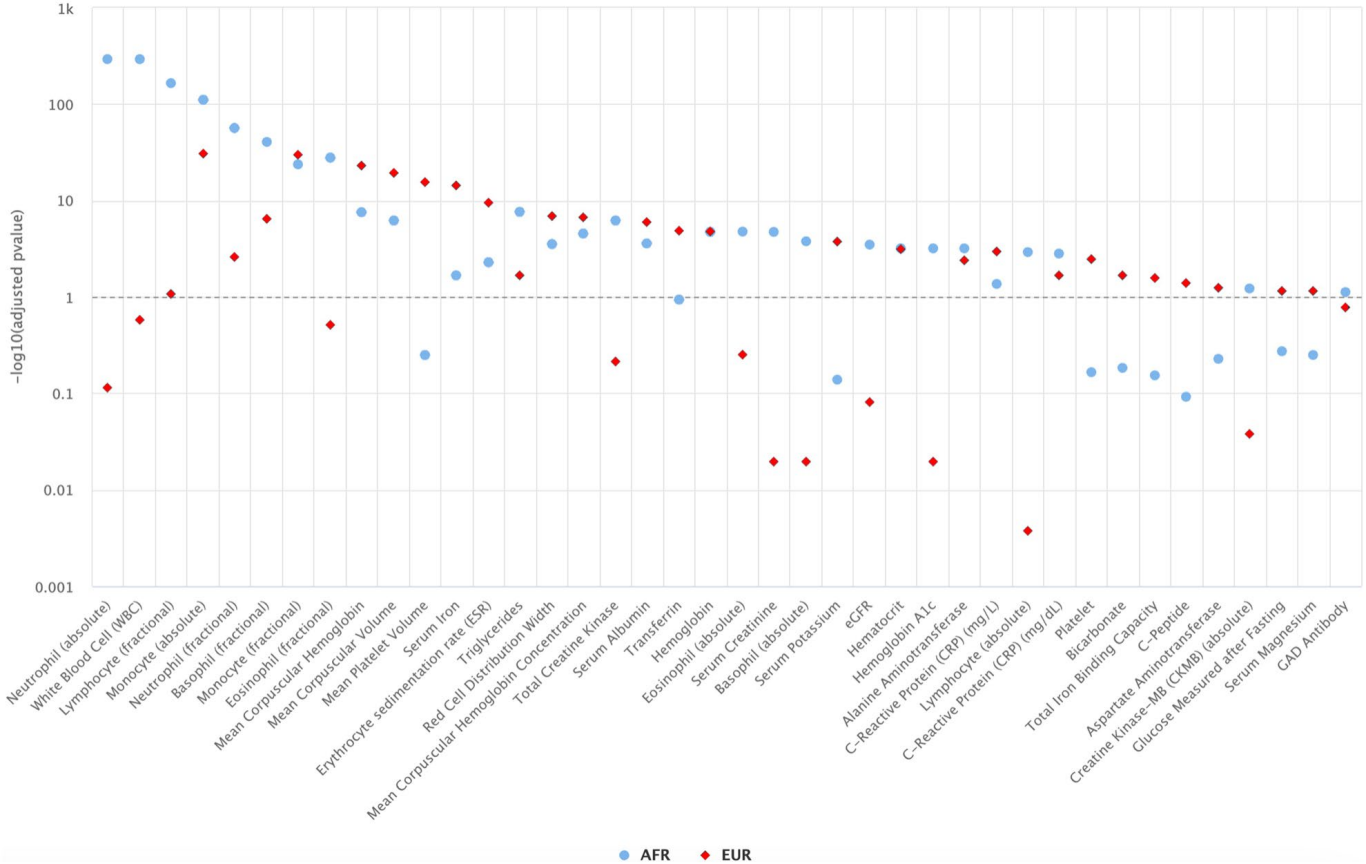
Laboratory measurements significantly associated with the *IL6R* variant in AFR or EUR (BH adjusted p value ≤ 0.1).

Overall, among phenotypes defined by PheCodes, we observed 10 with significant associations with *IL6R* among Veterans of AFR ancestry compared to 34 among Veterans of EUR ancestry, none of which were significant in both populations (Fig. 1). For laboratory measurements, we observed 30 measurements with significant associations with *IL6R* among Veterans of AFR ancestry compared to 28 among Veterans of EUR ancestry (Fig. 2). *IL6R* was significantly associated with 18 labs across both ancestries. As a positive control, based on prior knowledge of both the variant and the biologic function of blocking IL-6, we observed the expected association between the variant with lower C-reactive protein (CRP) and higher hemoglobin levels^4,31,32^ in both the EUR and AFR populations (Table S1).

The strongest associations within AFR subjects were related to white blood cell count (WBC), specifically, elevated WBC odds ratio (OR) 1.2, 95% confidence interval (CI), 1.1–1.3 (Fig. 1) by PheCode. The majority of *IL6R*-phenotype associations within EUR subjects pertained to vascular and cardiac disease. The phenotypes with the strongest association with *IL6R* were aortic aneurysm (AA) (OR 0.92; 95% CI, 0.90–0.94) as well as a specific type of aortic aneurysm, abdominal aortic aneurysm (AAA) (OR, 0.89; 95% CI, 0.87–0.90), coronary atherosclerosis and ischemic heart disease (CHD) (OR, 0.96; 95% CI, 0.95–0.97) (Fig. 1). The corresponding associations in AFR were similar but not significant [(AA) OR = 0.95 (0.87–1.03); (AAA) OR = 0.89 (0.80–1.00); (CHD) OR = 0.99 (0.95–1.02)].

**Figure 3:**
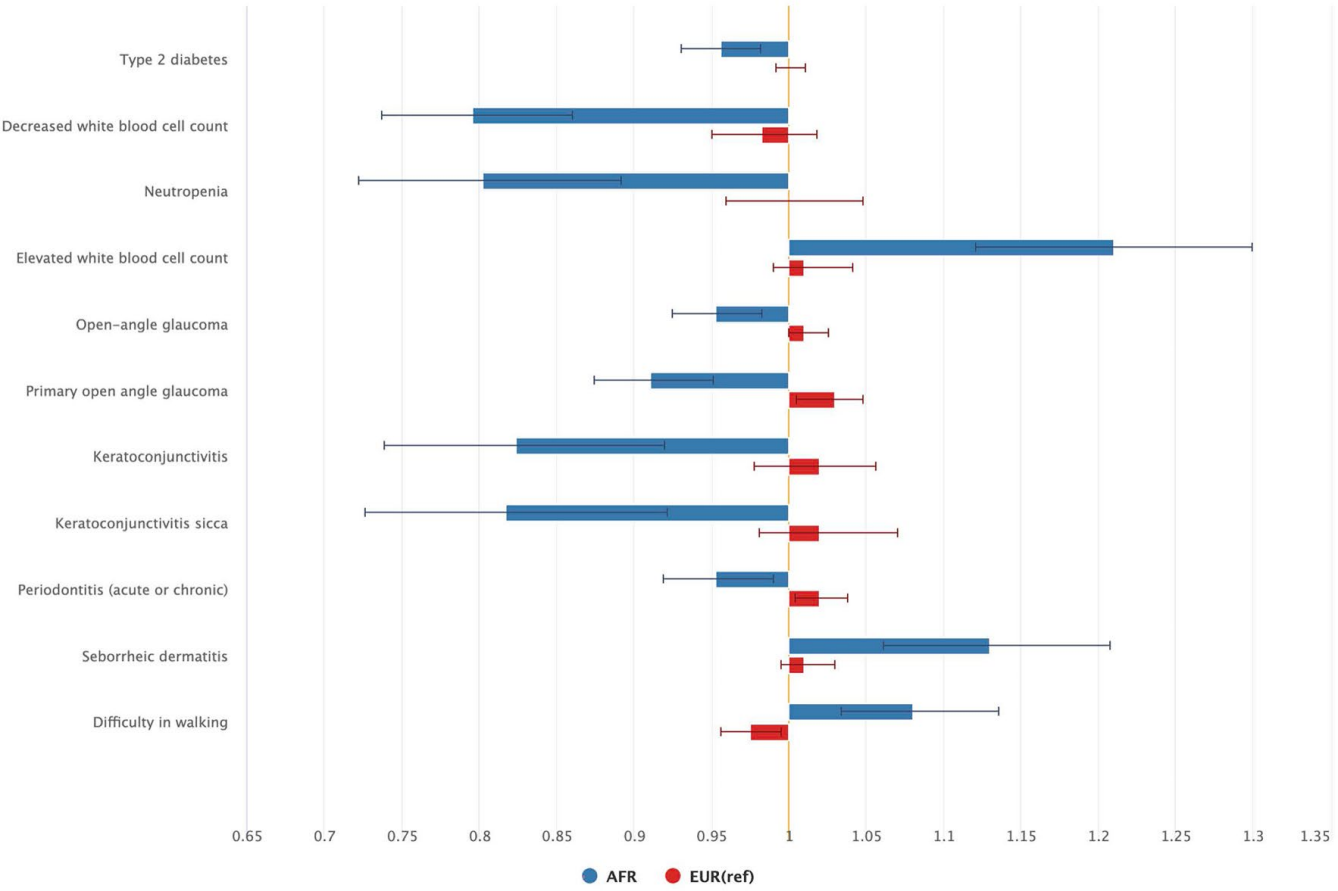
Odds ratios for phenotypes with significant differential associations in AFR vs EUR ancestries (BH adjusted p value ≤ 0.1), see also Supplementary Table S2.

After applying the test for heterogeneity, we observed 11 PheCodes translating to 7 conditions with differential association in AFR vs EUR: glaucoma, keratoconjunctivitis, periodontitis, type 2 diabetes, seborrheic dermatitis, walking difficulties, white blood cell count elevation (Fig. 3 and Table S2). *IL6R* was associated with reduced odds for glaucoma, keratoconjunctivitis, periodontitis, and type 2 diabetes among AFR with either no association or increased odds in EUR. The *IL6R* variant was associated with higher odds of an elevated white blood cell count in AFR (OR 1.21, 95% CI 1.12–1.30), and in line with this, a lower odds ratio for neutropenia in AFR (OR 0.80, 95% CI 0.72–0.89); these associations were not observed among EUR. *IL6R* was associated with seborrheic dermatitis and difficulty walking with increased odds in AFR and reduced odds in EUR.

**Figure 4:**
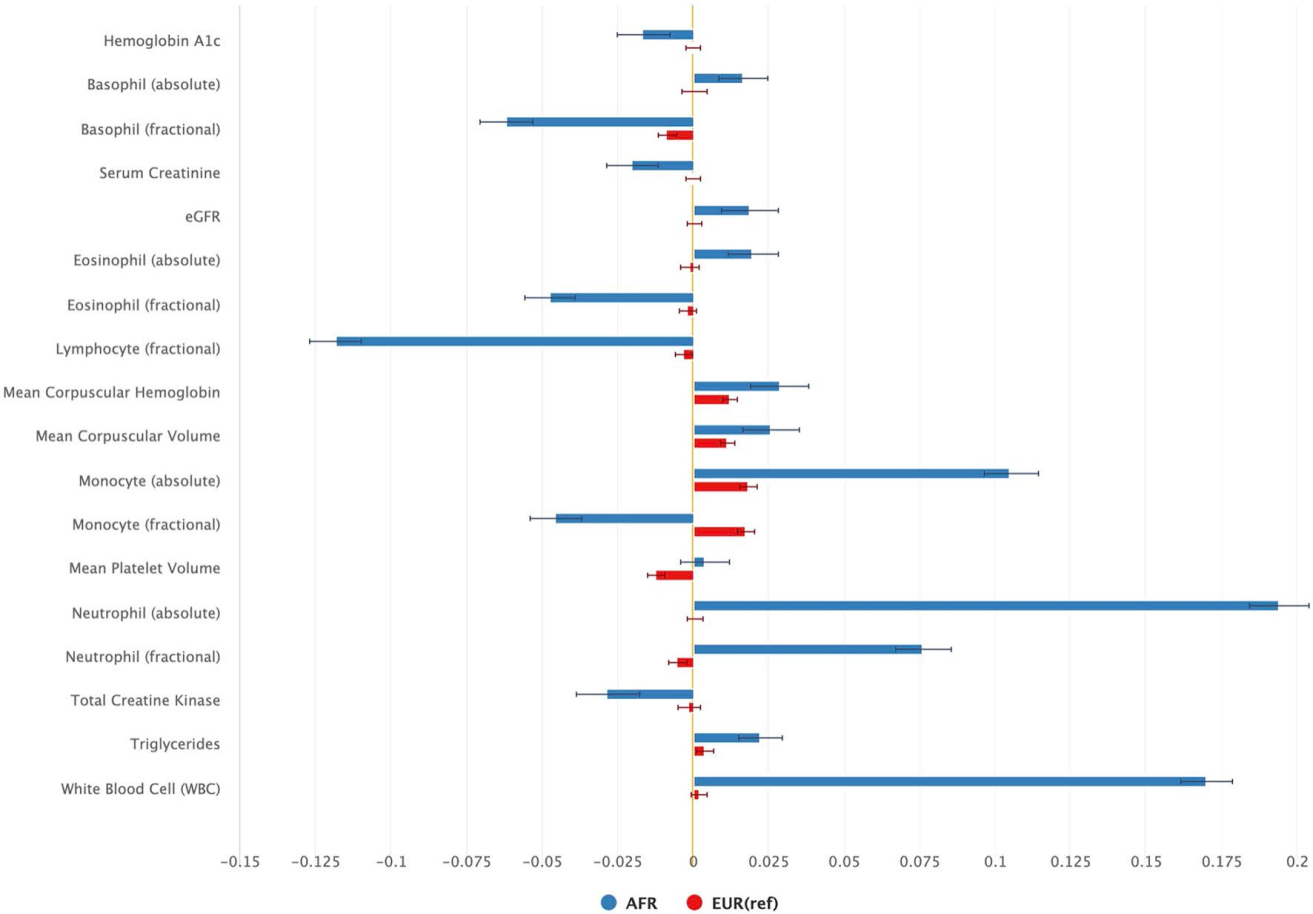
Comparison of standardized coefficients for associations between *IL6R* with laboratory values in AFR vs EUR (BH adjusted p value ≤ 0.1), see also Supplementary Table S3.

A comparison of laboratory values identified differences across 18 laboratory measurements (Fig. 4 and Table S3). In line with the significant difference in ICD codes related to WBC, the largest difference was observed in WBC whereby among individuals of AFR ancestry, each copy of the *IL6R* variant was associated with a higher WBC compared to those who did not carry the variant; no association was observed between *IL6R* and WBC among EUR. The higher value was observed across neutrophils, monocytes, eosinophils, and basophils, with the difference was most pronounced in absolute neutrophil count; the *IL6R* variant was associated with higher absolute values of neutrophils in AFR vs EUR. *IL6R* was also associated with higher triglyceride levels in AFR compared to EUR. The variant was associated with lower hemoglobin a1c (hba1c) in AFR with no significant association observed in EUR, in line with a lower odds ratio of T2D observed in AFR.

Due to the limited cohort size of individuals of AFR ancestry in the UKB and MGB, validation was focused on replicating laboratory values. The association and differences in WBC in AFR vs EUR remained the most significant finding. *IL6R* was associated with higher WBC among individuals of AFR vs EUR in both cohorts (Tables S4 and S5). *IL6R* was also associated with higher triglycerides in AFR vs EUR across the replication cohorts.

To understand the potential implications of the differential associations between *IL6R* with white blood cell phenotypes, we further tested the association between the variant and serious infection stratified by ancestry^33^. Overall, we observed an association between *IL6R* and a modest but significantly increased odds of serious infection in AFR but not EUR [AFR OR 1.03, 95% CI 1.01–1.04 vs EUR with OR 1.01, 95% CI 1.00–1.01]. Due to the small population size in UKB and MGB we did not have sufficient power to validate in these populations.

## Discussion

This study provides a new roadmap for leveraging large biobanks to screen for differential associations between genetic variants and phenotypes across a diverse population. These data in turn can be used to inform potential differential effects of targeted therapies using an application designed to test for heterogeneity in large-scale genotype–phenotype screens to complement or inform clinical trials where populations are smaller and more homogeneous. We focused on a specific variant in *IL6R* with the known downstream effect of reducing IL-6 signaling with effects similar to existing therapies targeting IL-6R.

In this study using the most recent data from MVP, a biobank with the largest population of individuals of AFR ancestry to date, we observed 29 traits with heterogeneous associations, including WBC and T2D. The most significant heterogeneous signal observed was a lower odds ratio of neutropenia or higher WBC among Veterans of AFR descent compared to EUR; in EUR no association was observed between *IL6R* and WBC. The clinical significance of the association between *IL6R* and higher WBC, particularly neutrophil counts in AFR and EUR ancestry is unclear. To provide context, in a large population-based epidemiologic study, WBC was lower in Black compared White individuals^34^. As WBC are involved in host defense, in the present study, we tested the association between *IL6R* and serious infection and observed a modest but significant increased odds for serious infection among individuals of AFR descent where no association was observed in EUR. We were underpowered to validate these findings in UKB or MGB. In a review of the literature, we were unable to identify clinical trials of therapies targeting IL6 stratifying outcomes or adverse events by self-reported race (as genetic ancestry data are typically not available in trials). The majority of large observational studies for infection risk and IL6R blockade stems from studies of tocilizumab, the first IL6R antagonist approved for use in the US for RA. In these studies, risk of infection on tocilizumab is compared with another targeted therapy and overall, no difference has been observed^33,35^, however there were no data stratifying by self-reported race or ethnicity. Proposed follow-up analyses of a published study stratifying by self-reported race were underpowered since only a subgroup of their data had available information on race and ethnicity^33,36^. Based on findings from the present study, we anticipate that in studies with adequately sized populations, we would anticipate higher WBC among individuals of AFR ancestry on IL6R blockade, as well as a potential small increased odds for serious infection compared to EUR. Future trials and studies on the IL6 pathway can consider collecting data on WBC and neutrophil count, as well as stratifying infectious adverse events by self-reported race.

The heterogeneity test also identified an association between the *IL6R* variant with a reduced odds of T2D among Veterans of AFR descent, while no association was observed in EUR. Likewise, hba1c which reflects an average level of glucose over 2–3 months, was lower among individuals of AFR carrying the *IL6R* variant, while no association was observed among EUR in MVP. A lower hba1c was also observed among AFR carrying the *IL6R* variant compared to EUR in UKB. To our knowledge, glucose and hba1c levels were not reported in the randomized controlled trials in rheumatoid arthritis or giant cell arteritis^37–39^. However, the general association between the *IL6R* variant and lower odds of T2D was observed in meta-analysis examining the potential role of this pathway in the etiology of T2D^40,41^. Additionally, higher serum IL6 levels are associated with higher levels of hba1c, and increased risk of developing T2D in a large cohort study of women^41,42^. In an observational cohort study of RA patients with hba1c measurements before and after initiation of tocilizumab compared to a tumor necrosis factor inhibitor, a larger reduction in hba1c was observed in the tocilizumab group^43^. Thus, our study corroborates these findings and further anticipates that individuals of AFR descent either with T2D or at risk of T2D may derive more benefit from IL6R compared to individuals of EUR descent.

Notably, the strong associations observed between the *IL6R* variant and cardiovascular phenotypes, e.g. coronary heart disease, aortic aneurysms, peripheral arterial disease observed in prior studies was confirmed in EUR but not AFR^6,17,44^. This difference in association between *IL6R* and cardiovascular phenotypes in AFR vs EUR did not reach statistical difference with regards to heterogeneity. The hetFDR approach leverages information from both the mean effect and the magnitude of heterogeneity to determine the significance of the differences based on data from the entire population. Thus, in comparison to other phenotypes studied, the differential association with CV phenotypes were not considered heterogeneous and we would not anticipate a significant difference in the salutary effect of IL6R blockade for CV phenotypes in AFR vs EUR.

The hetFDR procedure applied in this study for multiple testing of heterogeneity fills an unmet need for methods that allow us to screen high-throughput data efficiently, such as PheWAS for differences across diverse patient populations. Compared with existing commonly used FDR control approaches such as BHq^13^ and Storey’s procedure^28^, our method is more powerful in detecting the phenotypes with heterogeneous effects. HetFDR takes advantage of the fact that among all phenotypes, only a small fraction has non-zero effects and nearly all those phenotypes with heterogeneous effect tend to have non-zero mean effects on the whole population, which can be characterized more effectively compared to the heterogeneity due to the larger sample size. This property was confirmed with our simulation results given in the Supplementary Materials. Specifically, we demonstrated in a simulation study using a similar scale of data and variable types as our current biobank datasets, the hetFDR achieved a satisfactory FDR control and a uniformly higher power compared to other existing methods. Lastly, in our study we use IL6R as an example, however, multiple other genetic variant-drug pairs exist that can benefit from further subgroup analysis. For example, studies on the proprotein convertase subtilisin/kexin type 9 (PCSK9) inhibitors identified an increased risk of type 2 diabetes, diastolic blood pressure, type 1 diabetes, peptic ulcer disease, and depression^45^.

Finally, we note that the IL6R Asp358Ala allele is of particular interest because the biochemical profile of subjects with this variant is similar to subjects receiving IL6R antagonist therapy. However, the precise mechanism of action differs. The IL6R variant leads to reduced expression on membrane-bound IL6R while, the IL6R antagonists tocilizumab and sarilumab block both soluble and membrane-bound IL6R. This highlights that these methods and the use of PheWAS to investigate potential drug effects are meant to generate hypotheses. Follow-up studies are needed to determine whether the potential heterogeneity is present among subjects actually on treatment.

### Limitations

The population sizes for individuals of AFR ancestry were significantly lower in the UKB and MGB biobanks compared to MVP (UKB, AFR: n = 7,538; EUR: n = 459,315; MGB, AFR: n = 2922; EUR: n = 49,883; MVP, AFR: n = 105,838; EUR: n = 439,309). The smaller population resulted in limited power to replicate binary phenotypes, e.g., phecodes. Another potential limitation or difference between UKB and MVP is that UKB primarily contains inpatient codes and data from general medicine practices with less capture from other outpatient specialty practices in comparison to MVP and MGB. Importantly, this study did not include individuals of other ancestries.

This study focused on rs2228145, a relatively well-characterized loci and examined in prior studies as a potential proxy for IL6 blockade^4,17,18^. However, the majority of studies on genetic risk were performed in individuals of EUR ancestry, thus raising concerns regarding whether differences could be due to issues such as LD patterns. Given the limited existing data available regarding this locus in the AFR population, we believe this locus remains the best candidate to test for heterogeneity for the following reasons. In an eQTL mapping study in EUR and AFR populations, the top hit identified for IL6R was rs4846525^46^. We identified that this SNP had a D′ of 1.0 with rs2228145 in AFR and EUR populations. Additionally, the anticipated biologic associations, lower CRP and higher hemoglobin was observed in AFR, thus confirming the known and expected downstream functional effects in both the AFR and EUR populations.

## Conclusion

In summary, we leveraged 3 large population-based biobanks and applied a novel approach to test for heterogeneity identifying differential associations of the *IL6R* variant in AFR compared to EUR ancestry. Since the effect of the *IL6R* variant on phenotypic traits is known to parallel the effects of existing therapies targeting IL6R, findings from this study can inform ongoing and future trials targeting this pathway in the general population, particularly CVD. Our results suggest that targeting IL6R may be associated with higher WBC count and a potential modest signal for higher infection risk among individuals of AFR vs EUR descent. IL6R blockade may have a more beneficial effect for T2D with lower hba1c levels in AFR vs EUR, as well as potential beneficial effects for glaucoma, keratoconjunctivitis, and periodontitis. Notably, we observed a paucity of clinical trial data that were either sufficiently powered or reported data enabling post-hoc analyses of potential differences in effect across race and ethnicity. The increasing data available from more diverse populations such as MVP, along with the advancements in methods to analyze these data, can provide either complementary data or guidance on data elements to collect for pre-planned clinical trial subgroup analyses. Ultimately, these data together with approaches such as hetFDR can help us to design efficient trials that are powered to study the effectiveness of not just the primary outcome, but also potential beneficial and detrimental effects of a given therapy across a diverse population.

### Supplementary Information

Supplementary Information.

## Data Availability

The data that support the findings of this study are available from the corresponding author upon reasonable request.
