# Supplementary material for: Heterogeneous associations between interleukin-6 receptor variants and phenotypes across ancestries and implications for therapy
Source: Sci Rep. 2024 Apr 5;14:8021. doi: 10.1038/s41598-024-54063-3 (PMC10997791; doi:10.1038/s41598-024-54063-3)
Supplement: Supplementary file 1 — Supplementary Information. [file 41598_2024_54063_MOESM1_ESM.docx]

# Supplementary Materials

**Table S1. Significant and similar associations (with adjusted p-values) between the IL6R variant with CRP and hemoglobin in AFR and EUR.**

|  | **AFR** | | | **EUR** | | |
| --- | --- | --- | --- | --- | --- | --- |
| **Laboratory measurement** | **ES** | **SE** | **Adjusted pval** | **ES** | **SE** | **Adjusted pval** |
| **CRP mg/dL, median** | -0.0590 | 0.0171 | 5.56E-04 | -0.0185 | 0.0068 | 6.53E-03 |
| **CRP mg/L median** | -0.0522 | 0.0220 | 1.77E-02 | -0.0240 | 0.0066 | 2.60E-04 |
| **Hemoglobin g/dL, median** | 0.0032 | 0.0007 | 3.88E-06 | 0.0011 | 0.0002 | 3.27E-06 |

**Table S2. Significant heterogeneous associations (with adjusted p-values) between the IL6R variant and phecode based phenotypes in MVP in AFR vs EUR, reduced odds in yellow, increased odds in purple. (OR=odds ratio)**


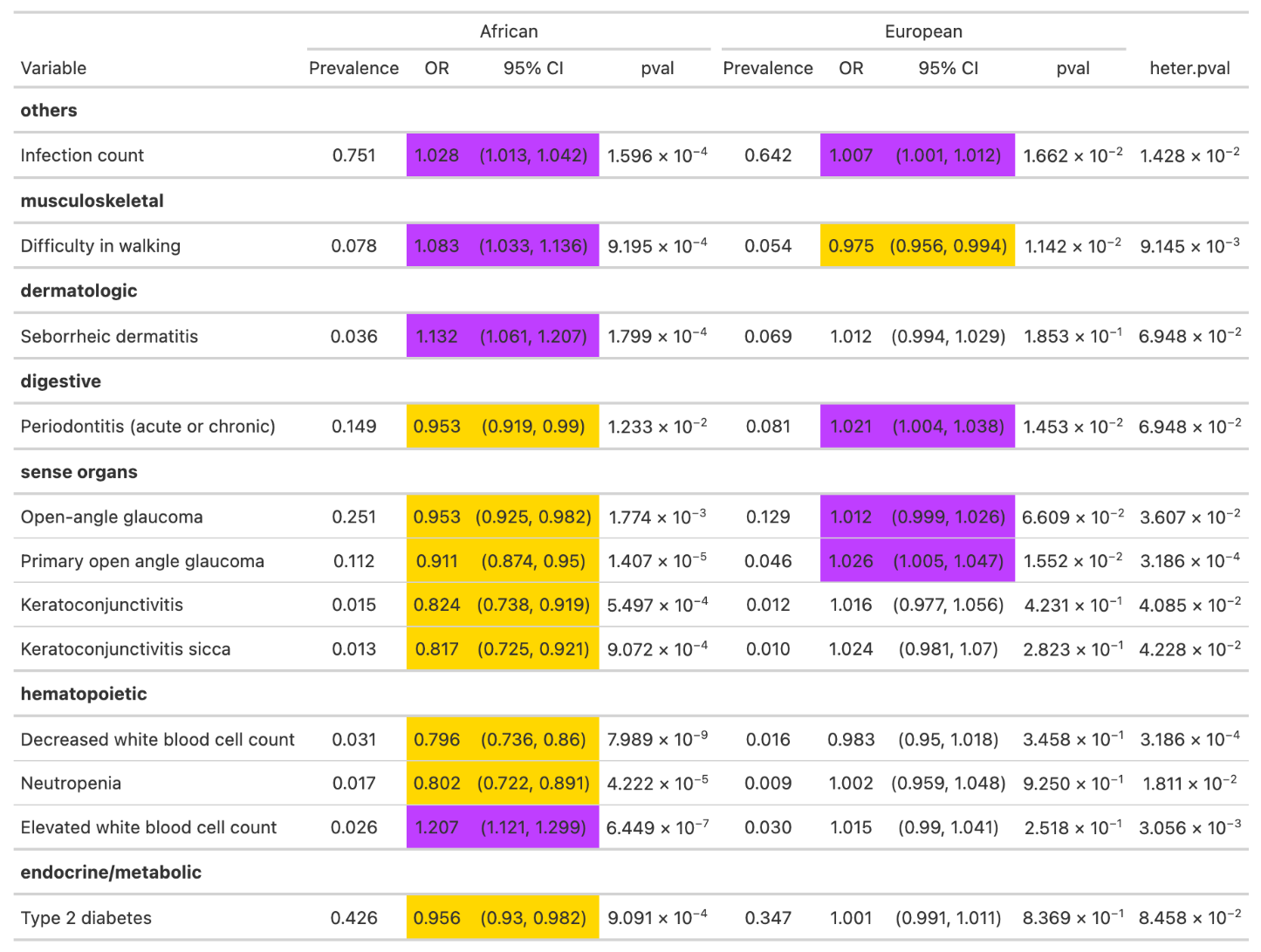


**Table S3. Significant heterogeneous associations (with adjusted p-values) between IL6R with median laboratory values in AFR vs EUR ancestry in MVP; comparisons with heterogeneous p-value< 0.1 were considered significant, negative association in yellow, and positive association in purple. (ES=effect size)**


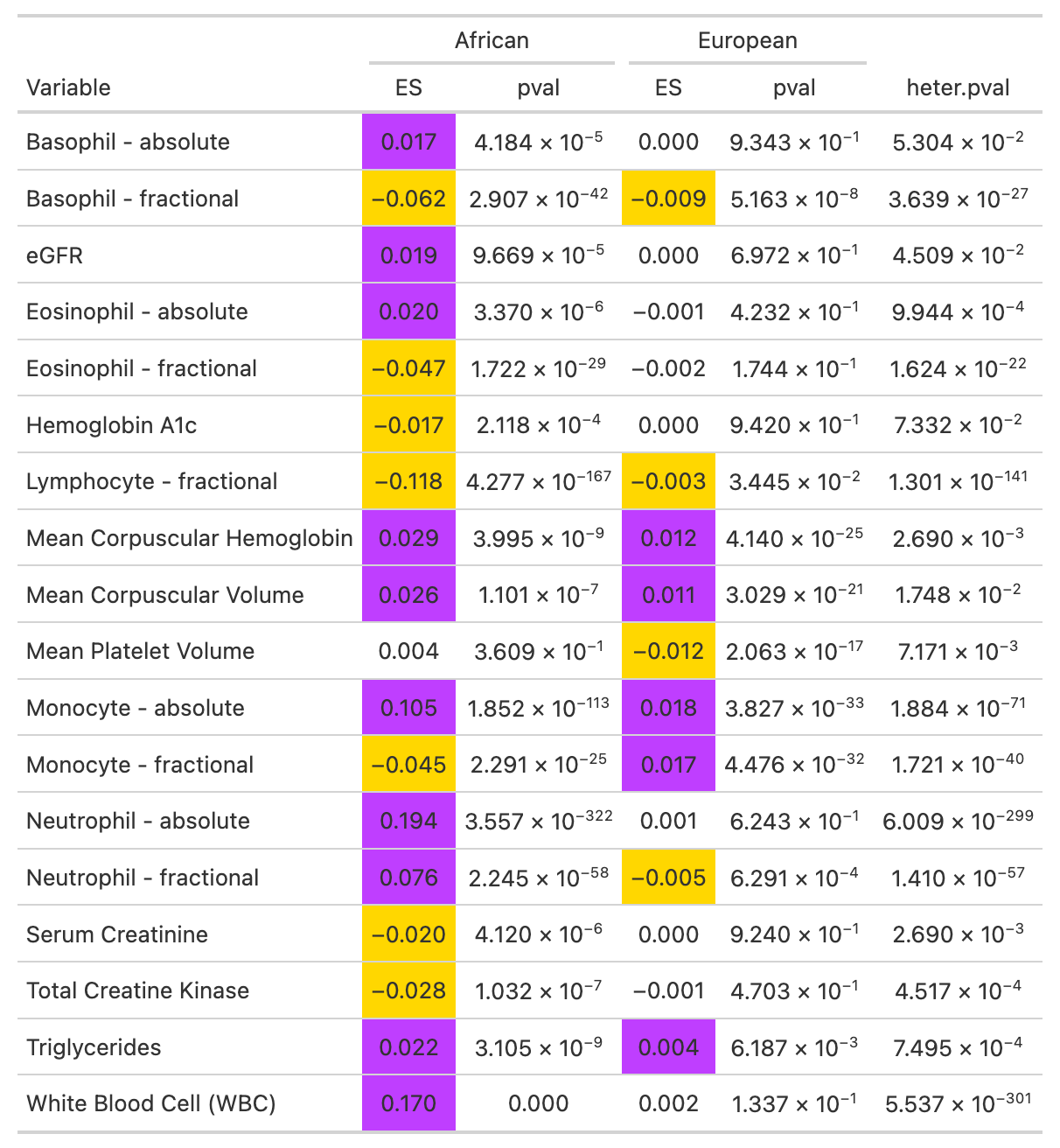


**Table S4. Validation results in the MGB Biobank for significant heterogeneous laboratory findings in AFR vs EUR in MVP (ES=effect size).**


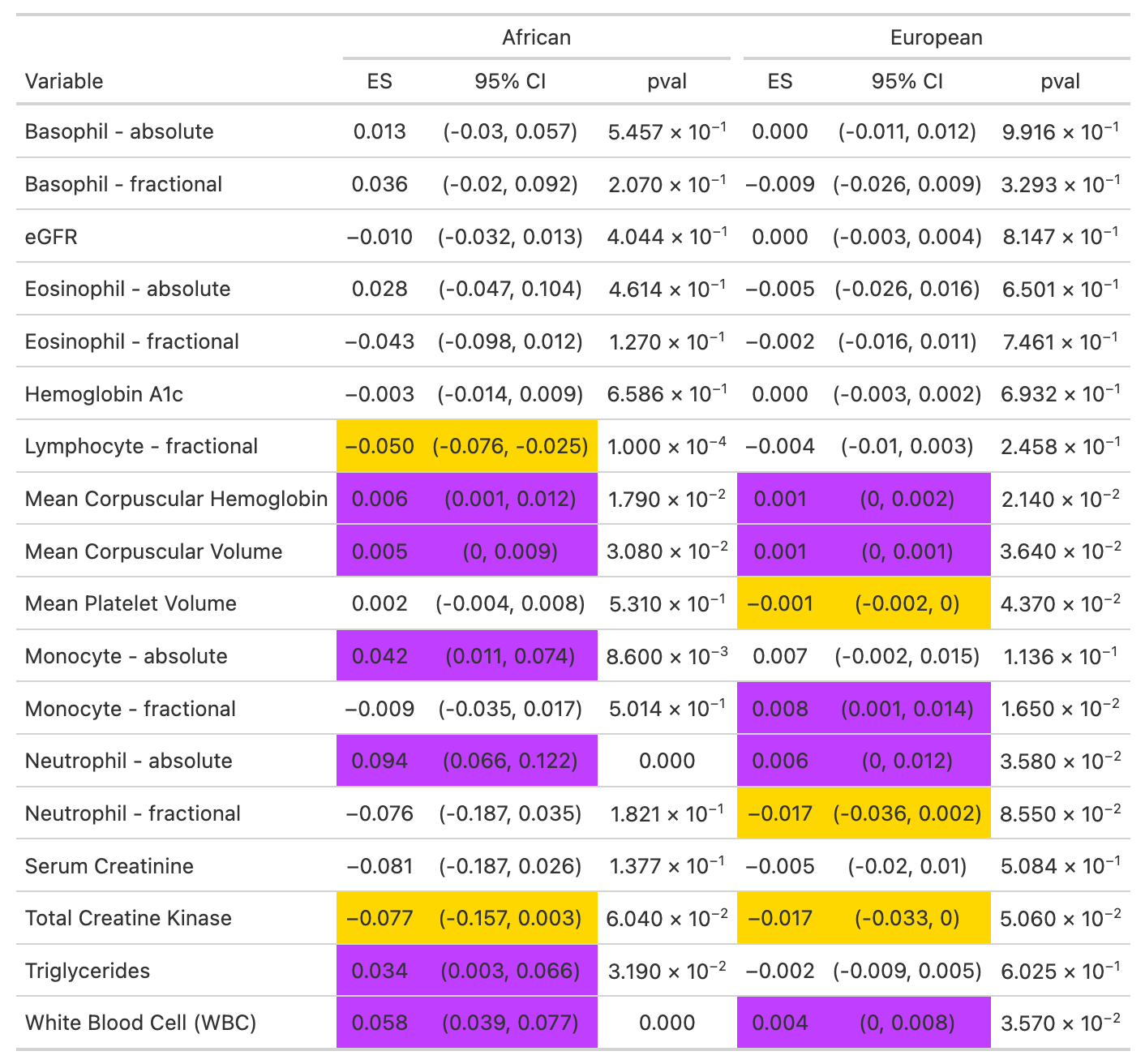


**Table S5. Validation results in UKB for significant heterogeneous laboratory findings in AFR vs EUR in MVP (ES=effect size).**


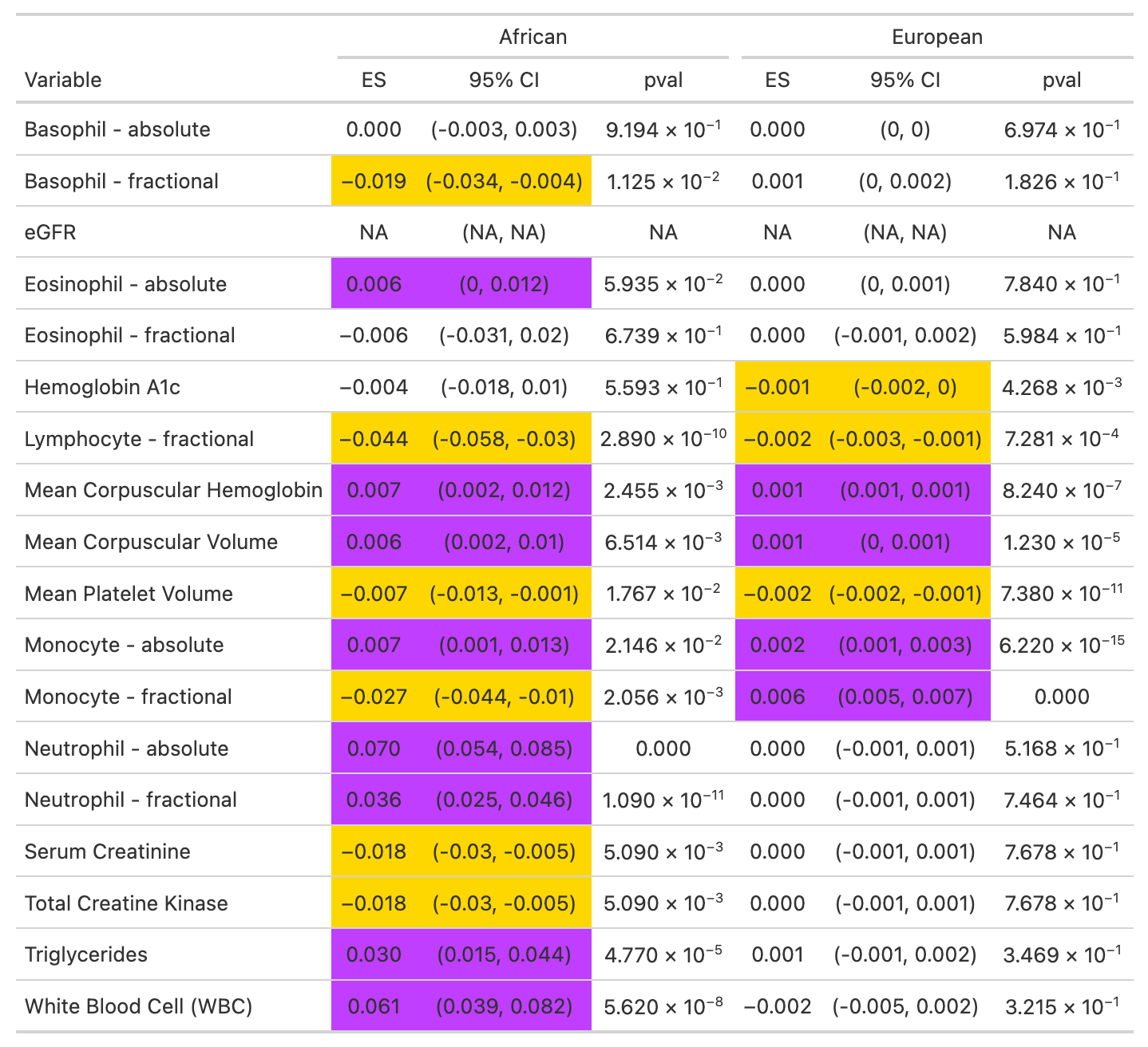


**Table S6. Curated laboratory measurements available in MVP (n=69).**

| **Description** | **Description** |
| --- | --- |
| Alanine Aminotransferase | Mean Corpuscular Hemoglobin |
| Aspartate Aminotransferase | Mean Corpuscular Hemoglobin Concentration |
| Basophil - absolute | Mean Corpuscular Volume |
| Basophil - fractional | Mean Platelet Volume |
| Beta-Hydroxybutyrate | Monocyte - absolute |
| Bicarbonate | Monocyte - fractional |
| Blood Glucose | Neutrophil - absolute |
| Blood Urea Nitrogen | Neutrophil - fractional |
| Brain Natriuretic Peptide | Platelet |
| B-Type Natriuretic Peptide (ProBNP) | Prostate Specific Antigen (PSA) |
| C-Peptide | Prothrombin Time (PT) |
| C-Reactive Protein (CRP) (mg/L) | Red Blood Cell(RBC) |
| C-Reactive Protein (CRP) (mg/dL) | Red Cell Distribution Width |
| Creatine Kinase-MB (CKMB) - absolute | Rheumatoid Factor (RF) |
| Creatine Kinase-MB (CKMB) - fractional | Serum Albumin |
| Cyclic Citrullinated Peptide Antibodies | Serum Calcium (mg/dL) |
| eGFR | Serum Calcium (mmol/L) |
| Eosinophil - absolute | Serum Chloride |
| Eosinophil - fractional | Serum Creatinine |
| Erythrocyte sedimentation rate (ESR) | Serum Iron |
| Ferritin | Serum Magnesium |
| GAD Antibody | Serum Potassium |
| Glucose Measure by Fingerstick | Serum Sodium |
| Glucose Measured after Fasting | Soluable Trasferrin Receptor |
| HDL-C | Total Cholesterol |
| Hematocrit | Total Creatine Kinase |
| Hemoglobin | Total Iron Binding Capacity |
| Hemoglobin A1c | Transferrin |
| Hepatitis C Lab Tests, Hepatitis C branched-DNA viral load test | Triglycerides |
| Hepatitis C Lab Tests, Hepatitis C RNA viral load (VL) test | Troponin Subtype I |
| International Normalized Ratio | Troponin Subtype T |
| LDL-C | Troponin without specified subtype |
| Lipoprotein-A | Uric Acid |
| Lymphocyte - absolute | White Blood Cell (WBC) |
| Lymphocyte - fractional |  |

**Statistical Method****ology**

**False discovery rate (FDR) controlled heterogeneity testing (hetFDR)**

In this section, we present the implementation details of our proposed false discovery rate (FDR) controlled heterogeneity testing (hetFDR) approach.

**Notation and Setup**

Let $J$ be the number of ancestry groups, and $K$ be the number of outcomes in association testing (phenotypes or laboratory values). For each subject $i$ belonging to the ancestry group $j\in\{1,2,\ldots,J\}$ with $n_{j}$ subjects, let $\mathbf{Y}_{i}^{\left( j \right)}=\left( Y_{i1}^{\left( j \right)},Y_{i2}^{\left( j \right)},\ldots,Y_{iK}^{\left( j \right)} \right)^{T}$ be the $K$-dimensional binary outcome vector of subject $i$, $\mathbf{X}_{i}^{\left( j \right)}=\left( 1,X_{i1}^{\left( j \right)},X_{i2}^{\left( j \right)},\ldots,X_{ip}^{\left( j \right)} \right)^{T}$ be the $\left( p+1 \right)$-dimensional adjustment covariates (e.g. age, and gender) vector including $1$ for intercept, and $A_{i}^{\left( j \right)}$ be the exposure variable (i.e. in this study the IL6R variant). Let

$$\mathcal{D}^{\left( j \right)}=\left\{ \mathbf{D}_{i}^{\left( j \right)}=\left( \mathbf{Y}_{i}^{\left( j \right)},A_{i}^{\left( j \right)},\mathbf{X}_{i}^{\left( j \right)}: i=1,\ldots,n_{j} \right) \right\}$$

denote the data set of each ancestry group $j$. To characterize the association between $Y_{ik}^{\left( j \right)}$ and $A_{i}^{\left( j \right)}$, we introduce the logistic model:

$$P\left( Y_{ik}^{\left( j \right)}=1|A_{i}^{\left( j \right)},\mathbf{X}_{i}^{\left( j \right)} \right)=g\left( \beta_{k}^{\left( j \right)}A_{i}^{\left( j \right)}+\gamma_{k}^{\left( j \right)T}X_{i}^{\left( j \right)} \right),$$

where $g\left( a \right)=e^{a}/\left( 1+e^{a} \right)$ represents the logistic link function. For phenotypes $k=1,2,\ldots,K$, we aim at simultaneously testing for heterogeneity of the effect $\beta_{k}^{\left( j \right)}$ across the $J$ ancestry groups:

$$\mathcal{H}_{0,k}:\beta_{k}^{\left( 1 \right)}=\cdots=\beta_{k}^{\left( J \right)}\text{ v.s. }\mathcal{H}_{1,k}:\beta_{k}^{\left( j_{1} \right)}\neq\beta_{k}^{\left( j_{2} \right)}\text{ for some }j_{1},j_{2}\in\{1,\ldots,J\},$$

with the false discovery rate (FDR) controlled below some level $\eta$ (e.g., $\eta=0.1$):

$$E\left[ \frac{\text{\#of false discovery}}{\text{Total \#of discovery}} \right]\leq\eta.$$

**Constructing Test Statistics**

We first construct the effect estimator $\hat{\beta}_{k}^{\left( j \right)}$ for each $\left( k,j \right)$ and its asymptotic variance $\hat{\sigma}_{k}^{\left( j \right)}$ using the standard score test. Then for each phenotype $k$, we introduce a mean effect statistic constructed as the inverse-variance weighted average of $\hat{\beta}_{k}^{\left( j \right)}$ across ancestry groups:

$$\hat{\mu}_{k}=\frac{\sum_{j=1}^{J} \left( \hat{\sigma}_{k}^{\left( j \right)} \right)^{-2}\hat{\beta}_{k}^{\left( j \right)}}{\sum_{j=1}^{J} \left( \hat{\sigma}_{k}^{\left( j \right)} \right)^{-2}}\overset{\mathcal{H}_{0,k}}{\sim}N\left( 0,\frac{1}{\sum_{j=1}^{J} \left( \hat{\sigma}_{k}^{\left( j \right)} \right)^{-2}} \right),$$

as well as a heterogeneity statistic constructed as the sample variance of $\hat{\beta}_{k}^{\left( j \right)}$ among ancestry groups:

$$\hat{T}_{k}=\sum_{j=1}^{J} \left( \hat{\beta}_{k}^{\left( j \right)}-J^{-1}\sum_{j_{1}=1}^{J} \hat{\beta}_{k}^{\left( j_{1} \right)} \right)^{2}\overset{\mathcal{H}_{0,k}}{\sim}\sum_{j=1}^{J} \lambda_{k}^{\left( j \right)}\left( \chi_{k}^{\left( j \right)} \right)^{2},$$

where $\left( \chi_{k}^{\left( 1 \right)} \right)^{2},\ldots,\left( \chi_{k}^{\left( J \right)} \right)^{2}$ are independent chi-squared random variables with degree of freedom $1$, and $\lambda_{k}^{\left( j \right)}$’s are estimated by extracting the eigenvalues of the $J\times J$ empirical covariance matrix:

$$\hat{\Sigma}_{k}=\frac{\sum_{j} \left( \hat{\sigma}_{k}^{\left( j \right)} \right)^{2}}{J^{2}}\mathbf{1}_{J\times J}-\left[ \mathrm{diag}\left( \frac{\left( \hat{\sigma}_{k}^{\left( j \right)} \right)^{2}}{J} \right) \right]_{j=1}^{J}\mathbf{1}_{J\times J}-\mathbf{1}_{J\times J}\left[ \mathrm{diag}\left( \frac{\left( \hat{\sigma}_{k}^{\left( j \right)} \right)^{2}}{J} \right) \right]_{j=1}^{J}+\left[ \mathrm{diag}\left( \left( \hat{\sigma}_{k}^{\left( j \right)} \right)^{2} \right) \right]_{j=1}^{J},$$

with $\mathbf{1}_{J\times J}$ representing the $J\times J$ matrix of all ones. Here the heterogeneity test statistic $\hat{T}_{k}$ is actually a quadratic form of Gaussian random variables. Its corresponding $p$-value $\hat{p}_{\alpha,k}$ can be computed using the *CompQuadForm* package in **R** (de Micheaux, 2017). Our testing and multiple testing of heterogeneity is carried based on $\{\hat{p}_{\alpha,k}:k=1,2,\ldots,K\}$. Meanwhile, we extract the $p$-value of the mean effect statistics $\hat{\mu}_{k}$, denoted as $\hat{p}_{\mu,k}$, as a guiding information to improve the power of the multiple testing; see the next section for details. It is important to note that under our construction, $\hat{\mu}_{k}$ and $\hat{T}_{k}$ are asymptotically independent, which grants the validity of multiple testing with $\hat{p}_{\alpha,k}$ assisted by $\hat{p}_{\mu,k}$.

**Weights Construction**

We use $\hat{p}_{\mu,k}$ as a prior guidance to assign weights to $\hat{p}_{\alpha,k}$, based upon the prior assumption that the non-null set of heterogeneity effects is close to that of the mean effects. Note that the effective sample size of $\hat{\mu}_{k}$ and $\hat{p}_{\mu,k}$ is the total sample size $\sum_{j=1}^{J} n_{j}$ of all ancestry groups, while that of $\hat{T}_{k}$ and $\hat{p}_{\alpha,k}$ is actually dominated by the minority ancestry groups with small sample size. Thus, $\hat{p}_{\mu,k}$ tends to provide a more precise information about the set of outcomes with non-zero mean effects, potentially serving as good side information to aid testing of the heterogeneity effects.

Inspired by recent literature of adaptive multiple testing that (Li and Barber, 2019; Cai et al., 2020, e.g.) leverages side guidance to enhance the power in comparison with the standard Benjamini Hochberg (BH) procedure (Benjamini and Hochberg, 1995), we propose the following procedures to convert $\hat{p}_{\mu,k}$ into proper weights of the candidate $p$-values $\hat{p}_{\alpha,k}$:

1. Calculate $Z_{\alpha,k}=I\left( \hat{p}_{\alpha,k}<\tau_{\alpha} \right)$ where $\tau_{\alpha}$ is some pre-specified cutoff parameter. Practically, one can either fix $\tau_{\alpha}$ as some small value like ${10}^{-4}$ or specify it empirically, e.g., choosing $\tau_{\alpha}$ as the $p$-value cutoff returned from the BH procedure on $\{\hat{p}_{\alpha,k}:k=1,2,\ldots,K\}$ with level $0.5$ (Cai et al., 2020).
2. Implement logistic regression on $Z_{\alpha,k}$ against $logit\left( 1-\hat{p}_{\mu,k} \right)=log\{\hat{p}_{\mu,k}/\left( 1-\hat{p}_{\mu,k} \right)\}$ to obtain the intercept $a_{0}$ and coefficient $a_{1}$. And set

$$\hat{\pi}_{k}=max\left\{ 1-\frac{1-exp\left\{ a_{0}+a_{1}logit\left( 1-\hat{p}_{\mu,k} \right) \right\}}{1-\tau},0 \right\}.$$

3. Standardize $\hat{\pi}_{k}$ and obtain the final weights $\hat{q}_{k}$ through: $\hat{q}_{k}=K\left( \sum_{k=1}^{K} \hat{\pi}_{k} \right)^{-1}\hat{\pi}_{k}.$

Our construction of $\hat{\pi}_{k}$ is motivated by the idea to find the non-null prior probability and the bayesian decision rule for each $k$ as used in Cai et al. (2020). And our third step to standardize $\hat{\pi}_{k}$ is used to protect the validity and FDR control.

**Adaptive FDR Control**

Finally, we weight and adjust the heterogeneity testing $p$-values as $\hat{p}_{\alpha,k}^{q}=min\{\hat{p}_{\alpha,k}/\hat{q}_{k},1\}$ for $k=1,2,\ldots,K$, and implement the following algorithm for discovery with FDR control of level $\eta$.

Algorithm 1. Adaptive multiple testing with FDR level $\eta$.

1: Find $\hat{k}=max\left\{ k\geq1:\hat{p}_{\alpha,k}^{q}\leq\left( k\eta/K \right)\wedge\tau_{\alpha}\text{ for at least }k\text{ many }\hat{p}_{\alpha,k}^{q} \right\};$

2: Reject null hypothesis $\mathcal{H}_{k}$ with $\hat{p}_{\alpha,k}^{q}\leq\left( \hat{k}\eta/K \right)\wedge\tau_{\alpha}$ for a total of $\hat{k}$ rejections.

**Simulation Studies**

We carried out simulation studies to evaluate hetFDR and compared it with commonly used FDR control approaches including Benjamini and Hochberg’s procedure (BHq) (Benjamini and Hochberg, 1995) and Stoery’s procedure (Storey, 2002). Each of our simulated dataset has 1000 phenotypes and two ancestry groups. We consider two different setups on the sample sizes of the two ancestry groups. One is a balanced sampling setup with 500 subjects in each group, and the other is imbalanced with 500 subjects in group 1 and 2000 in group 2. Note that the size ratio 1:4 in this imbalanced setting is close to that between AFR and EUR in the real application. The set of non-null mean effects $S_{\mu}=\{1,2,\ldots,50\}$ is of size 50 and that of heterogeneous effects $S_{\alpha}$ is set as the first $\left| S_{\alpha} \right|$ indices in $S_{\mu}$ so that $S_{\alpha}\subseteq S_{\mu}.$ For each ancestry group $j\in\{1,2\}$, we generate the exposure $A^{\left( j \right)}$ from $\text{Bernoulli}$(0.5), i.e., Bernoulli distribution with mean 0.5, and the covariates $X^{\left( j \right)}$ of dimensionality three from independent normal distribution. For phenotypes $k$ belonging to the null set, we generate outcome $Y_{k}^{\left( j \right)}\sim\text{Bernoulli}\left\{ \text{expit}\left( \gamma^{T}X^{\left( j \right)} \right) \right\}$ where $\text{expit}\left( u \right)=e^{u}/(1+e^{u}),$ and $\gamma=\left( 0.25,0.25,0.25 \right)^{T}$. For $k\in S_{\mu}\backslash S_{\alpha}$, i.e., phenotypes with non-zero mean effects and zero heterogeneity effects, we generate $Y_{k}^{\left( j \right)}\sim\text{Bernoulli}\left\{ \text{expit}\left( 0.4A^{\left( j \right)}+\gamma^{T}X^{\left( j \right)} \right) \right\}$. For phenotypes belonging to the heterogeneity set, i.e. $k\in S_{\alpha}$, we take $Y_{k}^{\left( j \right)}\sim\text{Bernoulli}\left\{ \text{expit}\left[ \left( 0.4-\alpha\right)A^{\left( j \right)}+\gamma^{T}X^{\left( j \right)} \right] \right\}$ for $j=1$ and $Y_{k}^{\left( j \right)}\sim\text{Bernoulli}\left\{ \text{expit}\left[ \left( 0.4+\alpha\right)A^{\left( j \right)}+\gamma^{T}X^{\left( j \right)} \right] \right\}$ for $j=2$ where the parameter $\alpha$ characterizes the strength of heterogeneity effects. We implement two set of simulation settings. Firstly, fix $\alpha=0.3$ and let the number of heterogeneous effects $\left| S_{\alpha} \right|$ vary in $\{10,25,40\}$. Second, fix $\left| S_{\alpha} \right|=40$ and let $\alpha$ vary in $\{0.2,0.3,0.4\}$. The desirable FDR level is set as $0.1$ and the FDR and average power of all methods are estimated via $500$ times of simulations in each setting.

The resulted FDR and average power under the balanced sample size setting (500 in both groups) are presented in Figure S1. Under different settings of the effect magnitude $\alpha$ and the number of heterogeneous effects $\left| S_{\alpha} \right|$, our proposed hetFDR method controls FDR below $0.1$ and shows substantial and consistent higher average power than BHq and Storey’s procedures. For example, when $\left| S_{\alpha} \right|=25$ and $\alpha=0.3$, hetFDR has about $0.3$ higher power than the other two methods. This is because that our method additionally leverages the mean effect statistics as side information and assigns higher chances of rejection to the phenotypes belonging to $S_{\mu}$. Since $S_{\alpha}\subseteq S_{\mu}$, this successfully reduces the price of screening out a large number of phenotypes with null effects. One may also note that our method is conservative on FDR control when $\left| S_{\alpha} \right|$ becomes larger. As an example, its FDR is around $0.05$ when $\left| S_{\alpha} \right|=40$, much smaller than the nominal level $0.1$ achieved by the other methods. We believe this is due to that our weighting procedure can significantly restrict the candidate set to $S_{\mu}$ by largely down-weighting the $p$-values of phenotypes with significant mean effects. Then when $S_{\alpha}$ takes large proportion ($80\%$ when $\left| S_{\alpha} \right|=40$) in $S_{\mu}$, hetFDR will be conservative since it is a BH-type procedure using the total number of hypotheses (effectively close to $\left| S_{\mu} \right|$ in our method) to approximate the number of false discoveries.

In Figure S2, we presented the results of the setting with imbalanced sample sizes (500 v.s. 2000). Again, FDR is well-controlled by all methods and our method has significantly larger power than both BHq and Storey’s methods across all the settings on $\left| S_{\alpha} \right|$ and $\alpha$. Compared to the balanced setting, all methods show moderately higher powers due to the increased sample size (from 500 to 2000) in one subgroup. Interestingly, the power enhancement of hetFDR over BHq or Storey’s method also becomes more prominent in the imbalanced setting than the balanced one. For example, our method has around 0.4 higher power than BHq and Storey when $\left| S_{\alpha} \right|=10$ in the former setting while having only 0.2 higher power in the latter one. This is because larger sample size of the majority group can lead to more accurate characterization of the overall effect, which in turn provides more effective prior information to improve the power of the heterogeneity testing in our method.

**Figure S1. FDR and average power plotted against different choices of α or |Sα| under the balanced sample size (500, 500) setting. Methods under comparison include our proposed hetFDR, the standard BHq procedure, and Storey’s procedure. The results are generated based on 500 times of simulations.**

**Figure S2. FDR and Average power plotted against different choices of α or |Sα| under the setting of imbalanced sample sizes (500, 2000). Methods under comparison include our proposed hetFDR, the standard BHq procedure, and Storey’s procedure.**

**References**

1. Benjamini, Y. and Hochberg, Y. (1995). Controlling the false discovery rate: a practical and powerful

approach to multiple testing. Journal of the Royal statistical society: series B (Methodological), 57(1):289-300.

2. Cai, T. T., Sun, W., and Xia, Y. (2020). Laws: A locally adaptive weighting and screening approach to

spatial multiple testing. Journal of the American Statistical Association, pages 1-30.

3. de Micheaux, P. L. (2017). Package compquadform. CRAN Repository.

4. Li, A. and Barber, R. F. (2019). Multiple testing with the structure-adaptive benjamini-hochberg algorithm.

Journal of the Royal Statistical Society: Series B (Statistical Methodology), 81(1):45-74.

5. Storey, J. D. (2002). A direct approach to false discovery rates. Journal of the Royal Statistical Society: Series B (Statistical Methodology), 64(3):479498.
